# Disintegration of Default Mode: Functional MRI Insights Into N,N-Dimethyltryptamine (DMT), Consciousness, and Subcortical Connectivity

**DOI:** 10.7759/cureus.88931

**Published:** 2025-07-28

**Authors:** Amy Avakian

**Affiliations:** 1 Department of Radiology, Southern Hills Hospital, Las Vegas, USA; 2 Department of Radiology, Ross University School of Medicine, Miramar, USA

**Keywords:** default mode network (dmn), dmt, fmri neuroimaging, functional mri (fmri), pituitary, pituitary dmt dmn

## Abstract

N,N-Dimethyltryptamine (DMT), a potent endogenous psychedelic, evokes rapid and immersive shifts in consciousness that challenge conventional neurocognitive models. Functional magnetic resonance imaging (fMRI) and electroencephalography (EEG)-fMRI studies suggest that these altered states are not chaotic but patterned, marked by a disruption of the default mode network (DMN), an increase in global integration, and heightened subcortical activity. Under the influence of DMT, the brain becomes less modular and more fluid, reorganizing itself into a hyperconnected state. Activity increases in the thalamus, amygdala, and hippocampus, regions involved in memory, emotion, and sensory salience. Meanwhile, the cortical regions typically responsible for self-referential processing lose their organizing control. This temporary collapse of hierarchical brain structure is associated with vivid imagery, emotional release, and a loosening of the boundaries that define the self. Rather than simply distorting perception, DMT appears to reveal the brain’s capacity to restructure consciousness itself. These findings suggest that consciousness may not be a fixed entity housed within a singular network but a flexible process shaped by context, neurochemistry, and meaning.

## Introduction and background

N,N-Dimethyltryptamine (DMT), a powerful endogenous psychedelic compound, has long challenged conventional models of consciousness. Structurally related to serotonin and naturally occurring in mammals, DMT induces rapid-onset, short-duration altered states marked by vivid visual hallucinations, ego dissolution, and profound shifts in time, identity, and emotional tone. While such experiences were historically relegated to mysticism or anecdote, advances in neuroimaging have begun to capture their biological underpinnings with increasing clarity.

At the center of this altered state lies an enduring question: What is the biological basis of consciousness, and to what extent can it be pharmacologically modulated? The pineal gland, long considered a neurospiritual locus in both ancient philosophy and modern speculation, remains a compelling target for inquiry, especially given the presence of DMT-synthesizing enzymes such as indolethylamine-N-methyltransferase (INMT) in both peripheral and central tissues.

Recent work using simultaneous electroencephalography (EEG) and functional magnetic resonance imaging (fMRI) has revealed that DMT significantly disrupts the brain’s default mode network (DMN), a system central to self-referential thought and the continuity of egoic identity [1]. This disintegration is accompanied by increased signal diversity, reduced alpha and beta oscillatory power, and a surge in global brain integration. Under DMT, the brain becomes less modular and more entropic, entering a hyperconnected state in which usual hierarchies collapse and previously segregated systems begin to synchronize [1].

These neural changes correlate with the phenomenology of the DMT experience: immersion in vivid imagery, emotional intensity, time distortion, and a loosening or dissolution of the self. As top-down predictive processing breaks down, bottom-up sensory and emotional input asserts greater influence, resulting in a state of consciousness that feels both unbounded and deeply personal. Subcortical regions such as the thalamus, hippocampus, and amygdala become unusually active, potentially explaining the archetypal visions and emotional catharsis often reported.

This paper synthesizes current neuroimaging evidence on DMT’s effects, with a focus on its disruption of the DMN and enhancement of subcortical connectivity. We propose that these alterations are not incidental distortions but reflections of a deeper plasticity within human consciousness, one that may help redefine our understanding of identity, perception, and the self.

## Review

The default mode network (DMN)

The default mode network (DMN) is a large-scale, functionally interconnected network in the human brain that activates preferentially during periods of rest and internally directed cognition. It is most active when individuals are not engaged in externally focused, goal-directed behavior but are instead immersed in reflection, memory recall, future planning, or spontaneous thought. While originally identified as a “task-negative” network, it is now widely recognized as the neural basis for self-referential processing, personal identity, and mental simulation [1,2].

Structural Anatomy of the DMN

The DMN consists of both midline and lateral cortical regions that demonstrate synchronized low-frequency activity during rest. These include the medial prefrontal cortex (mPFC), posterior cingulate cortex (PCC), and precuneus, along with the angular gyrus, lateral temporal cortex, and hippocampal formation. The mPFC is primarily associated with self-appraisal and the internal monitoring of one’s emotional and cognitive states, while the PCC and precuneus are involved in consciousness integration and autobiographical memory retrieval. The angular gyrus plays a role in semantic processing, narrative construction, and theory of mind, the ability to model the perspectives of others [1].

This network is neither anatomically localized nor isolated. Rather, it functions as a system of distributed cortical hubs whose activity is highly correlated during internally focused states. These hubs are functionally connected by white matter tracts, and their activity shows strong coherence in the low-frequency band (~0.01-0.1 Hz) as seen in resting-state fMRI [2].

Functional Roles and Narrative Integration

Far from being idle, the DMN is critically involved in maintaining a sense of self across time. It enables mental time travel, the ability to revisit personal memories and simulate possible futures. This autobiographical function helps construct and maintain a continuous identity, giving rise to the subjective experience of being “me” [1]. The network also supports internal narration, daydreaming, and moral reasoning, all of which contribute to the shaping of social behavior and personality [2].

This activity is not limited to the present moment. The DMN bridges past, present, and future through a process of narrative integration, whereby the brain constructs a cohesive story from lived experiences. The capacity to evaluate “who I was,” “who I am,” and “who I might become” relies heavily on DMN function and its communication with memory centers such as the hippocampus [1,2].

Anticorrelation and Dynamic Competition

When attention shifts from internal thoughts to external tasks, the DMN typically deactivates. This process reflects a dynamic interplay between the DMN and task-positive networks, such as the dorsal attention network and the central executive network. These systems are often anticorrelated, meaning when one becomes more active, the other becomes less so [1]. The ability to toggle between these modes, from introspection to action and from reflection to execution, is a critical feature of adaptive cognition.

Clinical Relevance: Hyperconnectivity and Hypoconnectivity

DMN dysregulation has been implicated in a wide range of clinical conditions. Hyperconnectivity within the DMN, particularly between the mPFC and PCC, is frequently observed in depression and anxiety, where it correlates with ruminative thinking and excessive self-focus. In contrast, hypoconnectivity or the fragmentation of DMN coherence is observed in schizophrenia and Alzheimer’s disease, where it may contribute to impairments in memory, identity continuity, and reality testing [2].

These opposing patterns of dysregulation suggest that the DMN operates within a delicate balance. When this balance is disrupted, the resulting distortions in internal narrative processing may manifest as psychological distress or cognitive fragmentation. Thus, the DMN serves as both a mirror of mental life and a therapeutic target for interventions aimed at restoring coherent self-processing.

Psychedelics and brain connectivity

Classic serotonergic psychedelics, including psilocybin, LSD, and N,N-dimethyltryptamine (DMT), have reemerged at the forefront of neuropsychiatric research, not only for their powerful subjective effects but also for their reproducible and striking impact on large-scale brain networks. At the system level, one of the most consistent findings across neuroimaging studies is that these compounds simultaneously reduce the functional integrity of high-level cortical networks, particularly the default mode network (DMN), while increasing cross-network integration across typically segregated systems. This dynamic reconfiguration reflects more than transient neurochemical disruption; it may represent a fundamental shift in how the brain processes and prioritizes information [2-4].

The DMN is crucial to maintaining the narrative self, introspective thought, and internal modeling of identity and time. Under the influence of psychedelics, the integrity of this network weakens, especially the connectivity between the medial prefrontal cortex (mPFC) and the posterior cingulate cortex (PCC), the two core hubs most consistently implicated in self-referential processing. Across studies, a common pattern emerges: psychedelics reduce intranetwork coherence within the DMN while increasing functional connectivity between the DMN and other brain networks, such as sensorimotor, salience, visual, and limbic regions [2,3,5].

In a comprehensive review of neuroimaging studies, classic psychedelics were found to consistently reduce DMN integrity and disrupt higher-order network structure while simultaneously enhancing communication between low-level sensorimotor regions and higher-order associative circuits. This reorganization supports a model of consciousness that becomes more globally interconnected under psychedelics, with less rigid control from top-down hierarchical systems [2].

This breakdown of functional segregation does not reflect unstructured noise or neural chaos. Rather, it reflects a predictable, ordered shift toward a brain state characterized by high entropy-greater signal diversity, increased flexibility, and reduced dominance of hierarchical network control. Psychedelics increase the overall entropy of the brain’s activity, allowing for a broader range of potential mental states to arise. The result is greater neural complexity and the emergence of patterns that are not ordinarily accessible in typical waking consciousness [3,6].

Importantly, this increase in entropy does not lead to functional impairment. Instead, it is associated with enhanced cross-network communication, improved emotional salience, and even increased global efficiency. Psychedelic-induced brain states, though less constrained, maintain coherent reorganization and exhibit greater capacity for integration. This reflects not a breakdown of cognition but an adaptive restructuring that temporarily releases the brain from its default patterns [4,6].

To conceptualize this phenomenon, the Relaxed Beliefs Under Psychedelics (REBUS) model has been proposed as a unifying theory. The model suggests that psychedelics temporarily reduce the influence of high-level predictions, or priors, within the brain’s hierarchical processing system. These priors normally shape and constrain how incoming information is interpreted. By relaxing them, psychedelics enable bottom-up sensory and emotional information to rise to conscious awareness with less filtering and distortion. This shift allows for new associations, insights, and perspectives to form [7].

This process of top-down suppression and bottom-up emergence has profound therapeutic implications. In mental health conditions such as major depressive disorder or post-traumatic stress disorder (PTSD), overly rigid beliefs and ruminative thought patterns are thought to result from maladaptive prediction models that become overly dominant. Psychedelics, by disrupting these pathological hierarchies, appear to enable emotional release, narrative reprocessing, and cognitive flexibility. The breakdown of rigid network hierarchies and the associated increase in global integration may underlie the clinical improvements observed in mood, cognition, and affect regulation following psychedelic treatment [2,7].

These therapeutic mechanisms are not unique to a single psychedelic compound. Despite variations in duration, intensity, and subjective content, psychedelics, including psilocybin, LSD, and DMT, share a remarkably consistent signature in their effect on network organization. They reliably disrupt the functional segregation of the DMN and other high-level systems while enhancing connectivity between networks involved in emotion, attention, and sensory processing [2,3,5].

This consistency across compounds reinforces the idea that the psychedelic state reflects a common system-level phenomenon. It supports the hypothesis that altered states of consciousness induced by psychedelics do not arise from nonspecific receptor binding alone but from coordinated shifts in system-level brain architecture [2,4].

Critically, the psychedelic brain state remains functionally viable and coherent. It is not a pathological state, such as that observed in delirium or psychosis, but one marked by increased responsiveness, reorganized coherence, and potential for reintegration. The psychedelic state is best understood not as a breakdown of function but as a metastable brain state: transient, flexible, and capable of yielding therapeutic value. It represents a window in which reorganization is possible and in which psychological rigidity may give way to insight and healing [2,6,8].

Taken together, the evidence suggests that psychedelics produce a dynamic reorganization of brain network architecture, centered on the breakdown of the DMN’s top-down control and the expansion of inter-network connectivity. This high-entropy, metastable state allows for flexibility, openness, and narrative deconstruction, qualities that may underpin their therapeutic potential. The richness of subjective experience under psychedelics, including ego dissolution, synesthesia, emotional breakthroughs, and enhanced perspective-taking, appears to mirror the brain’s move toward greater signal diversity and integration [2,3,4,7].

This neuroplastic and emotionally permissive state, while transient, may offer a unique opportunity for therapeutic intervention. When paired with psychological support and guided integration, the psychedelic experience becomes not merely an altered state but a potentially healing one, opening the door to reprocessing long-held beliefs, confronting repressed material, and fostering adaptive new frameworks of self and meaning [5,6,8].

DMT-Specific Findings

Among the classic serotonergic psychedelics, N,N-dimethyltryptamine (DMT) stands out for its rapid onset, short duration, and remarkable intensity. When administered intravenously, DMT produces a full psychedelic experience within seconds, peaks within two to three minutes, and resolves within 20 minutes. This condensed timeframe has made DMT a powerful tool for investigating the neurobiological basis of consciousness in real time using advanced imaging techniques such as EEG-fMRI [3,6].

Functional neuroimaging studies consistently show that DMT causes a rapid collapse of functional connectivity within the default mode network. The most prominent disruptions occur between the medial prefrontal cortex and the posterior cingulate cortex, which serve as central hubs of self-referential processing. These changes are accompanied by an increase in signal diversity and enhanced global integration across brain networks [6]. Simultaneous EEG recordings reveal a significant reduction in alpha oscillatory power, along with increased broadband signal diversity, which aligns with the subjective intensity of the experience [6,8].

During the DMT state, subcortical structures such as the thalamus, brainstem, and limbic system become more active and more connected to cortical regions. The thalamus, in particular, shows increased functional coupling with high-order association areas. This enhanced thalamocortical communication may account for the vivid sensory phenomena and heightened perceptual salience reported during DMT administration [6]. The limbic system, including the amygdala and hippocampus, also shows greater activation, consistent with emotional intensity, symbolic imagery, and autobiographical content emerging into awareness [6,8].

At the system level, the brain under DMT becomes less functionally segregated and more globally integrated. Functional boundaries between networks become more porous, allowing previously disconnected systems to interact in novel ways. This reorganization is associated with increased neural entropy, which refers to the greater variability and unpredictability of brain signals. Rather than reflecting cognitive instability, this high-entropy state allows for enhanced flexibility, openness to new associations, and dynamic adaptation [6].

Importantly, this state retains coherence and responsiveness. Although the usual narrative structure of consciousness may dissolve, individuals remain aware, lucid, and capable of reflecting on the experience afterward. The brain does not enter a state of dysfunction but rather enters a metastable mode of operation that facilitates novel insights and emotional breakthroughs [6,8].

DMT’s receptor binding profile contributes to these effects. Like other classic psychedelics, it is a potent agonist at the serotonin 5-HT2A receptor, which is widely distributed in cortical areas involved in cognition, perception, and emotion. In addition to its serotonergic action, DMT also binds to the sigma-1 receptor, a noncanonical intracellular receptor involved in calcium homeostasis, mitochondrial regulation, and neuroplasticity [3].

The sigma-1 receptor is known to function as a molecular chaperone at the interface between the endoplasmic reticulum and mitochondria. Its activation has been linked to reduced oxidative stress, the modulation of inflammatory responses, and protection against cellular damage. These functions may support neuronal stability during altered states of consciousness that require rapid energetic and regulatory shifts [3]. DMT’s engagement with the sigma-1 receptor may therefore contribute to the preservation of cognitive function and emotional processing during the intense sensory and emotional overload of the DMT experience.

This dual receptor activity distinguishes DMT from other psychedelics and may explain the paradoxical nature of its effects. Although users report the complete dissolution of the ego, boundary breakdown, and immersive visionary states, they do not describe cognitive fragmentation or the loss of consciousness. The experience is often described as organized, emotionally coherent, and deeply meaningful, despite its intensity and departure from ordinary awareness [6,8].

DMT may function not only as a temporary disruptor of conscious content but also as a modulator of the brain’s structural and functional organization. Through its effects on receptor systems that govern perception, affect, and intracellular signaling, DMT enables a transient but significant reconfiguration of large-scale neural dynamics. This state is characterized by a reduction in the brain’s reliance on top-down predictive control, an increase in bottom-up sensory and emotional input, and a global increase in network integration and signal complexity [3,6,8].

These neurophysiological features correspond closely with the core phenomenology of the DMT experience. Individuals report ego dissolution, the nonlinear perception of time, hyper-associative thinking, and intense emotional catharsis. Rather than emerging from randomness, these experiences appear to result from specific and measurable changes in brain activity that enable conscious access to otherwise inaccessible domains of mental functioning [6,8].

From a therapeutic perspective, these alterations in brain dynamics may facilitate the loosening of maladaptive cognitive and emotional patterns. By disrupting entrenched self-narratives and reactivating emotionally salient neural circuits, DMT may create a unique window for psychological flexibility and emotional reintegration. Although more research is needed, early findings suggest that DMT may hold clinical potential in the treatment of conditions characterized by rigid cognition, suppressed emotion, or traumatic memory loops [3,6].

**Figure 1:**
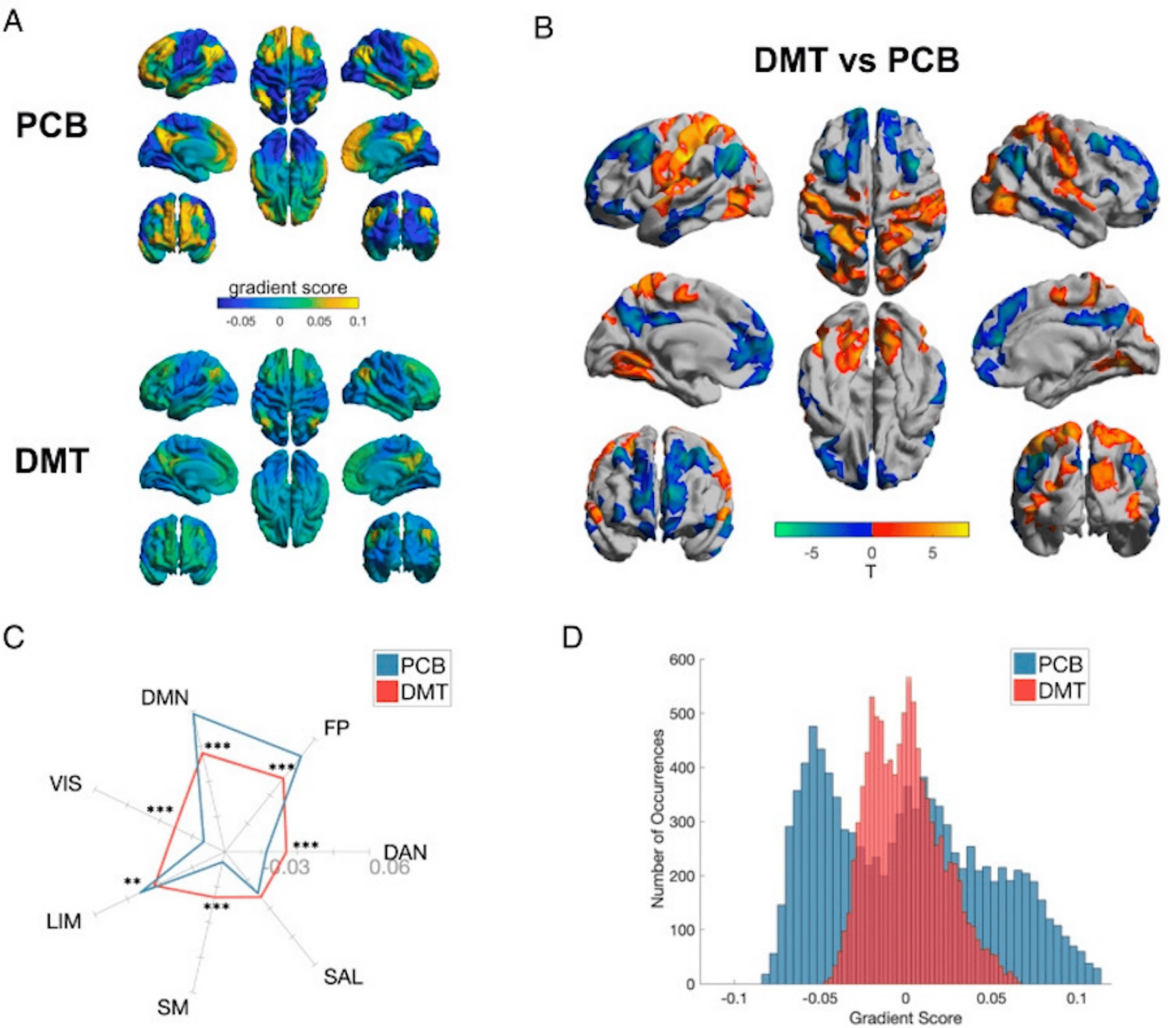
(A) Mean principal gradient for the placebo (PCB) (top) and DMT (bottom) conditions, representing the principal axis from the unimodal to transmodal cortex. (B) DMT > placebo between-group vertex-wise (top) and (C) network-wise (bottom) contrasts. Network-wise radial plot displays the mean intranetwork principal gradient score for each network for DMT and placebo (**P < 0.01 and ***P < 0.001; FDR corrected). (D) Histogram showing the distribution of principal gradient values for placebo and DMT conditions for each brain area. Adapted from Timmermann et al., 2023, licensed under CC BY 4.0 [8]. VIS, visual; SM, somatomotor; DAN, dorsal attentional; SAL, ventral attentional/salience; LIM, limbic; FP, frontoparietal; DMN, default mode network; FDR, false discovery rate; DMT, N,N-dimethyltryptamine

In summary, DMT produces a rapid and reversible reconfiguration of brain network architecture. It collapses the dominant role of the default mode network, enhances global signal diversity, engages subcortical and limbic regions, and enables dynamic cross-network integration. These effects reflect not a breakdown of function but a rebalancing of consciousness itself. Through this transient reorganization, DMT provides a window into the brain’s innate capacity for transformation, emotional processing, and cognitive liberation (Figure 1).

The Pineal Gland and Endogenous DMT

The pineal gland is a small neuroendocrine structure located near the center of the brain, nestled between the two thalamic hemispheres, and anatomically positioned within the third ventricle. It is evolutionarily conserved across vertebrates and is primarily recognized for its role in regulating circadian rhythms through the secretion of melatonin. Melatonin synthesis is tightly regulated by photoperiod input via the retinohypothalamic tract and the suprachiasmatic nucleus, linking external light cycles to hormonal modulation of sleep-wake patterns. While this function is well established, recent interest has reemerged regarding the pineal gland’s potential role in the endogenous biosynthesis of the hallucinogenic compound N,N-dimethyltryptamine (DMT), a serotonergic psychedelic known for its rapid onset, profound subjective effects, and unique pharmacological profile [3].

The hypothesis that DMT may be synthesized in the pineal gland stems from the identification of key biosynthetic enzymes within this region, particularly indolethylamine-N-methyltransferase (INMT). This enzyme catalyzes the methylation of tryptamine, a monoamine derived from the amino acid tryptophan, to form DMT. INMT has been found in a variety of mammalian tissues, including the retina, adrenal glands, spinal cord, and lung. Importantly, its expression has also been detected in the central nervous system, with some studies identifying INMT mRNA and protein in the pineal gland itself [3]. The presence of both INMT and its cofactor, S-adenosylmethionine (SAM), supports the biochemical plausibility of DMT synthesis within this brain structure.

Although the direct measurement of DMT levels within the pineal gland in humans remains technically and ethically challenging, rodent models have detected trace amounts of DMT in the brain, raising the possibility that it may be synthesized and released under specific physiological conditions. Some researchers have proposed that DMT functions as an endogenous neuromodulator that is produced in response to high-arousal states such as birth, near-death experiences, rapid eye movement (REM) sleep, or intense emotional or physical trauma [3]. These states share several neurophenomenological features, including vivid imagery, temporal distortion, ego boundary dissolution, and altered body awareness, which closely mirror the effects reported during exogenous DMT administration in experimental and clinical settings.

The anatomical and functional characteristics of the pineal gland add a compelling layer to this hypothesis. Its midline position, its connection to the visual system, and its regulatory role over endocrine rhythms place it at a unique intersection between sensory input, neurochemical release, and internal states of awareness. In addition, the pineal gland is unprotected by the blood-brain barrier, making it directly responsive to peripheral metabolic and hormonal signals. This anatomical accessibility raises the possibility that it could act as a neuroendocrine interface between environmental stimuli and internal conscious states.

From a system neuroscience perspective, the idea that DMT might be synthesized in the pineal gland and modulate brain network dynamics introduces the possibility that the brain possesses an intrinsic mechanism for rapidly altering consciousness. This would represent a form of neurochemical gating, in which the release of an endogenous hallucinogen triggers a cascade of alterations in cortical-subcortical communication, memory retrieval, emotional processing, and sensory integration. In particular, DMT’s affinity for 5-HT2A receptors, highly expressed in the prefrontal cortex and associative regions, may permit rapid top-down destabilization of default mode network (DMN) dominance, allowing access to alternative modes of self-processing. The concurrent engagement of the sigma-1 receptor, which is expressed in mitochondrial and endoplasmic reticulum membranes, may support cellular resilience during such entropic shifts by regulating calcium signaling and mitigating oxidative stress [3].

If DMT is indeed synthesized within the brain during altered states, it would join the ranks of endogenous neuromodulators such as serotonin, dopamine, and endorphins but with a functional role not in homeostasis or mood regulation per se but in consciousness modulation. This function may have evolved to serve adaptive purposes in facilitating neural reorganization during critical transitions such as parturition, trauma recovery, or the dream state. DMT’s ability to dissolve high-level cognitive priors, generate emotionally salient mental imagery, and alter time perception could allow for the processing of emotionally charged material that is inaccessible under normal waking cognition.

From a philosophical and symbolic standpoint, the association between the pineal gland and altered consciousness is not new. Historical texts across diverse cultures describe the pineal gland as a spiritual organ linked to clairvoyance, divine insight, and transcendent experience. René Descartes famously posited it as the seat of the soul, believing it to be the point of connection between the immaterial mind and the physical body. In Eastern traditions, the gland is often equated with the Ajna chakra, or “third eye,” a center of intuitive knowledge and inner vision. While such claims are metaphysical in origin, modern neuroscientific inquiry has begun to bridge these domains by revealing biologically plausible pathways through which the pineal gland may influence subjective experience via biochemical means [3].

Modern psychedelic research has demonstrated that exogenously administered DMT leads to consistent and reproducible changes in neural activity. These include reduced connectivity within the DMN, increased global brain integration, elevated signal complexity, and increased communication between limbic, sensory, and associative networks. Subjectively, these changes correspond to phenomena such as ego dissolution, emotional catharsis, spiritual insight, and narrative deconstruction. If endogenous DMT is capable of inducing similar effects in a state-dependent manner, it may contribute to specific forms of spontaneous psychological insight, mnemonic retrieval, or even visionary states that are deeply encoded in cultural and religious experience [3].

Despite the growing interest in the pineal-DMT hypothesis, significant gaps remain. There is no definitive proof of DMT production in the pineal gland under natural conditions, and in vivo detection remains technically limited by the extremely low concentrations involved and the rapid metabolism of the compound by monoamine oxidase enzymes. Moreover, the trigger conditions for any hypothetical endogenous release remain speculative. However, the presence of synthetic machinery, the similarity between spontaneous altered states and DMT-induced states, and the consistent effects observed in controlled settings provide a compelling rationale for continued investigation [3].

In summary, while the hypothesis that the pineal gland produces and releases DMT remains unproven, mounting biochemical and phenomenological evidence supports its plausibility. The anatomical uniqueness of the pineal gland, combined with the distribution of INMT and the receptor profile of DMT, suggests that endogenous psychedelics may play a role in modulating states of consciousness. Whether facilitating dream content, mediating peak spiritual experiences, or supporting neural flexibility in moments of transition, DMT may serve as a latent neurochemical pathway embedded within the architecture of the human brain. Continued research at this intersection of neurochemistry, consciousness science, and symbolic history may offer new insights into how the brain orchestrates the most mysterious aspects of subjective experience [3].

How we know what we know: Mapping the neural effects of DMT

The ability to study the brain under the influence of a powerful and fast-acting psychedelic compound such as N,N-dimethyltryptamine (DMT) has offered neuroscientists an unprecedented opportunity to examine the mechanisms of altered states of consciousness. In recent years, the combination of neuroimaging technologies with controlled psychedelic administration protocols has enabled researchers to explore real-time changes in brain network architecture with increasing precision. Through the use of functional magnetic resonance imaging (fMRI), electroencephalography (EEG), and hybrid EEG-fMRI recordings, scientists have begun to map the acute effects of DMT on both global and local brain activity, with a particular focus on large-scale networks such as the default mode network (DMN) and its interactions with sensory, affective, and associative systems [4-6,8].

Most of these studies involve the intravenous administration of DMT in carefully monitored clinical or laboratory settings. This route allows for precise control over the timing, dose, and onset of the psychedelic experience, which is essential for aligning subjective reports with neurophysiological recordings. The participants are typically fitted with EEG electrodes and simultaneously undergo fMRI scanning. This dual-modality approach permits the capture of both high temporal resolution (through EEG) and high spatial resolution (through fMRI), allowing researchers to observe rapid changes in brain activity and how those changes unfold across different regions over time [4,6].

In a typical study design, baseline resting-state scans are collected before DMT administration. Following intravenous injection, the participants remain still in the scanner while data are collected throughout the onset, peak, and resolution of the psychedelic state. Subjective experiences are often recorded using validated psychometric scales such as the Mystical Experience Questionnaire or the 11-Dimension Altered States of Consciousness scale. These self-reports are later compared with observed changes in brain network activity to explore how specific patterns of connectivity correlate with particular aspects of the experience, such as ego dissolution, emotional release, or vivid imagery [5,6].

To analyze the data, researchers employ a variety of computational techniques. Independent component analysis (ICA) is commonly used to identify functionally coherent networks based on shared patterns of blood oxygenation level‐dependent (BOLD) signal fluctuations. This method enables investigators to isolate intrinsic brain networks such as the DMN, salience network, dorsal attention network, and frontoparietal control network and assess how their internal coherence and between-network connectivity are altered under DMT. Seed-based correlation analysis, another widely used approach, allows for the selection of specific brain regions (seeds) and evaluates their connectivity with other regions across the brain. These techniques are particularly well suited to assess changes in functional segregation (i.e., how distinct a network remains from others) and integration (i.e., how well information is shared across networks) [4,5].

Several studies have reported that DMT leads to a breakdown in the integrity of the DMN, especially between the medial prefrontal cortex and posterior cingulate cortex, and enhances connectivity between typically segregated systems. These findings have been interpreted within the framework of increased global entropy, reduced top-down processing, and the opening of cross-modal associative pathways. Simultaneously, EEG recordings show significant reductions in alpha power and increases in broadband signal complexity, suggesting a more disordered and flexible neural landscape [6,8].

Notably, researchers have also focused on subcortical regions during DMT exposure. The thalamus, hippocampus, and amygdala, structures involved in sensory relay, emotional salience, and memory retrieval, show increased functional engagement, which may help explain the emotionally rich and symbolically intense features of the DMT experience. These areas also appear to communicate more extensively with high-order cortical regions during the psychedelic state, indicating that the brain enters a globally integrated mode in which the boundaries between systems are temporarily relaxed [6,8].

Ethical considerations are a central feature of modern psychedelic research, especially when working with powerful consciousness-altering compounds such as DMT. Protocols typically include rigorous participant screening to exclude individuals with a history of psychosis, bipolar disorder, or cardiovascular instability. All volunteers must provide informed consent and are given thorough briefings on potential risks and expectations. Sessions are conducted under medical supervision, often with trained psychological support staff present. Post-session integration is also standard practice, allowing participants to process and contextualize their experience in a safe and reflective setting [4].

Some of the most comprehensive and methodologically rigorous studies in this field have been conducted by leading researchers such as Robin Carhart-Harris, Christopher Timmermann, and Fernanda Palhano-Fontes. These investigators have played a foundational role in establishing both the technical standards and theoretical frameworks that currently guide psychedelic neuroscience. Their work has contributed to our understanding of how psychedelics affect large-scale brain networks, how subjective experiences relate to measurable brain states, and how these phenomena might be leveraged for therapeutic purposes [5,6,8].

As a result of these investigations, a clearer picture is beginning to emerge of how DMT alters the structure and function of consciousness. Through the use of multimodal imaging, sophisticated analytical techniques, and ethically grounded research practices, neuroscientists are increasingly able to track the trajectory of the psychedelic experience in the brain. The resulting data have not only deepened our understanding of DMT but also contributed to broader questions about the neural basis of consciousness, self-perception, emotional salience, and cognitive flexibility.

In summary, the mapping of DMT’s effects on the brain involves a carefully orchestrated interplay of technology, methodology, and human experience. Studies using fMRI, EEG, and advanced connectivity modeling have revealed consistent alterations in brain network dynamics that align closely with the phenomenology reported by participants. These findings provide a scientific framework for understanding how a single compound can produce such profound shifts in awareness and offer a foundation for future research into the therapeutic potential of psychedelics.

What Happens in the Brain During a DMT Trip

The acute effects of N,N-dimethyltryptamine (DMT) on the brain represent one of the most striking and reproducible models of altered consciousness in neuroscience. Unlike other psychoactive compounds that exert more diffuse or gradual influences, DMT induces rapid and profound changes in the functional architecture of the brain, generating experiences often described as immersive, symbolic, emotionally charged, and ineffable. The neurophysiological signatures of these experiences are becoming increasingly well defined through advances in neuroimaging and electrophysiological recording. Across multiple studies, DMT consistently produces a coordinated collapse of default network function, enhanced global signal diversity, the reorganization of subcortical processing, and the modulation of affective and integrative circuits in the brain [4-6,8].

One of the most robust findings across DMT studies is a rapid and significant disruption of the default mode network (DMN). This network includes the medial prefrontal cortex (mPFC), posterior cingulate cortex (PCC), precuneus, and lateral parietal cortex and is centrally involved in self-referential cognition, autobiographical memory, and the maintenance of narrative identity. The DMN serves as a kind of internal simulator, weaving together past experiences and future projections into a continuous sense of self [4].

During the DMT state, functional connectivity within the DMN decreases markedly. Timmermann et al. observed a breakdown in communication between core DMN hubs, particularly the mPFC and PCC, accompanied by reductions in alpha oscillatory power, which is often associated with quiet wakefulness and top-down control [6,8]. These changes parallel subjective reports of ego dissolution, boundary loss, and the suspension of self-referential thought. The participants often describe a complete disconnection from personal identity or temporally anchored memory, suggesting that the collapse of DMN coherence may play a causal role in producing non-ordinary states of awareness. The destabilization of the DMN is not merely a loss of signal but a redistribution of activity across other brain systems. As the self-referential core quiets, other networks, especially sensory, affective, and associative circuits, become more active and interconnected [4,6].

Concomitant with DMN disruption is a dramatic increase in functional connectivity between brain regions that are typically segregated under normal waking consciousness. This process, referred to as network desegregation or global integration, reflects a reorganization of information flow. Under DMT, communication between low-level sensory areas and high-order cognitive regions increases, and previously autonomous systems begin to share information in novel ways [6,8].

This heightened interconnectivity is accompanied by a measurable increase in signal entropy and complexity. EEG recordings during DMT administration consistently show a reduction in alpha power and a corresponding rise in gamma oscillations and broadband complexity. These signatures are interpreted as indicators of a more flexible and high-capacity brain state, allowing for new patterns of activity to emerge spontaneously [6].

Such states resemble the metastable dynamics seen during early development or creative insight, where the brain is not constrained by rigid priors but is able to explore multiple cognitive and perceptual trajectories. The reorganization of large-scale networks may underlie reports of heightened insight, emotional release, and expanded awareness during the DMT experience [6,8].

While cortical network dynamics have received the most attention, subcortical structures also play a critical role in shaping the DMT experience. Functional neuroimaging reveals increased activity in the thalamus, hippocampus, amygdala, and brainstem, regions involved in emotion, sensory gating, memory recall, and arousal regulation [6,8]. These systems, which are usually regulated by top-down cortical networks such as the DMN and frontoparietal control network, become more dominant during the psychedelic state.

The thalamus, in particular, appears to act as a gateway for sensory flooding. Under DMT, it shows enhanced connectivity with multiple cortical targets, which may account for the vivid visual, auditory, and somatic experiences reported by the participants. This increased thalamocortical coupling mirrors patterns seen during REM sleep and certain dreamlike states, suggesting that DMT places the brain in a hyper-associative, internally generative mode [6].

The amygdala and hippocampus also become more engaged during the DMT state, often correlating with emotional intensity and memory retrieval. This activity supports the interpretation that DMT may facilitate the emergence of emotionally charged or symbolic content, including themes of birth, death, transformation, and unity. Soares et al. demonstrated increased functional connectivity between the posterior supramarginal gyrus and regions involved in emotional regulation and social cognition, including the orbitofrontal cortex, precuneus, and PCC. Stronger coupling between the amygdala and orbitofrontal cortex further suggested enhanced affective salience and contextual sensitivity during the experience [5].

This rebalancing of subcortical influence may explain why DMT experiences often feel psychologically meaningful or cathartic, even in the absence of clear narrative continuity. Emotional material long buried in unconscious circuits may rise into awareness when cortical constraint is lifted, contributing to the therapeutic potential of the experience.

Time Perception, Narrative Collapse, and Self-Referential Rewiring

Another hallmark of the DMT state is the distortion or collapse of time perception. Many participants report that minutes feel like hours or that time disappears altogether. This subjective timelessness is mirrored in the loss of temporal structuring observed in DMN activity and the disruption of hippocampal-prefrontal coordination. The brain’s ability to sequence events, construct coherent storylines, and maintain episodic memory appears to falter during high-dose DMT exposure [6,8].

Instead of anchoring experience to personal history, the brain shifts into a state of pure presence, symbolic fluidity, and hyper-associative patterning. This loss of temporal coherence and narrative identity is often described as ego death or transpersonal awareness. The phenomenological signature includes merging with the environment, encountering archetypal beings, or perceiving a unity that transcends individuality. These states are consistently accompanied by decreased DMN coherence and increased activity in visual and emotional cortices [4,6].

Following the experience, many individuals describe lasting changes in how they perceive themselves and their relationships. This is consistent with the view that the psychedelic experience allows for a temporary rewiring of self-referential circuitry, creating a window of neuroplasticity in which old patterns may be released and new frameworks formed.

At the molecular level, the effects of DMT are initiated primarily through its agonist activity at the 5-HT2A receptor, a subtype of serotonin receptor abundantly expressed in layer V pyramidal neurons of the cortex. The activation of this receptor leads to increased excitatory postsynaptic currents and enhanced glutamatergic signaling, which in turn drive cortical desynchronization and the breakdown of hierarchical predictive coding [4,6].

However, DMT’s pharmacological profile is more complex than that of a typical serotonergic agonist. It also binds with high affinity to the sigma-1 receptor, an intracellular chaperone protein that modulates calcium signaling, mitochondrial activity, and neuroinflammatory responses. The sigma-1 receptor is known to play a role in protecting neurons from oxidative stress and regulating cellular adaptation to high-energy states. The activation of this receptor may help explain the structural and functional resilience observed in the brain during the highly entropic DMT state [8].

This dual action allows DMT to simultaneously destabilize cortical hierarchies and support cellular-level neuroprotection. The result is a state that is intense and destabilizing at the psychological level but not disruptive in the pathological sense. This combination may be key to its therapeutic potential, offering a kind of controlled cognitive breakdown paired with emotional reintegration. The subjective reports of DMT experiences, such as encountering intelligent entities, sensing profound interconnectedness, and receiving emotionally charged insights, are not easily explained through conventional neurocognitive models. However, when examined through the lens of network neuroscience and predictive processing, these phenomena begin to appear as emergent features of a brain operating under conditions of relaxed priors and increased bottom-up signaling.

As top-down constraints dissolve, sensory and emotional systems gain greater influence, leading to the spontaneous emergence of highly structured visions, symbolic narratives, and powerful affective responses. These patterns resemble dream logic, mythic imagery, and altered states described across religious and shamanic traditions. The neurobiological architecture uncovered in DMT studies offers a potential bridge between subjective mystical experience and empirical brain science. The enhanced entropy, signal diversity, and subcortical engagement observed during DMT exposure support a model in which consciousness is not fixed but metastable, capable of dynamic reorganization. In this model, the boundaries of self, time, and meaning are not static constructs but network-dependent illusions that can be dissolved and reconstructed through specific neuromodulatory processes [6,8].

Clinical Implications and Future Directions

The ability of DMT to transiently destabilize rigid self-narratives and facilitate emotional processing has profound implications for psychiatry. Conditions such as depression, PTSD, and addiction are often characterized by overly rigid cognitive structures, maladaptive prediction models, and persistent affective loops. By disrupting these loops and opening access to suppressed emotional content, DMT may serve as a powerful adjunct to therapeutic processes aimed at psychological reintegration.

Future research will need to clarify dose-response relationships, long-term effects, and the role of context in shaping therapeutic outcomes. Neuroimaging studies should continue to refine their temporal resolution and expand their integration with psychometric data, including qualitative interviews and longitudinal follow-up.

Additionally, understanding the conditions under which endogenous DMT may be synthesized or modulated could yield insights into the natural functions of altered states in human development and recovery. Whether DMT plays a role in dreaming, birth, or death remains speculative but testable with advances in biosensing and molecular neuroimaging.

The DMT experience is not a random or chaotic event in the brain. It reflects a coordinated shift in global network dynamics, molecular signaling, and psychological orientation. The collapse of default mode coherence, rise in global integration, reengagement of subcortical emotion centers, and modulation of self-processing circuits point to a reproducible neurobiological architecture of transformation. Through a temporary breakdown of rigid hierarchies, the brain enters a state of dynamic reorganization in which new meanings can emerge and old patterns can dissolve.

By studying DMT, researchers are not only unlocking the mechanisms of one of the most powerful altered states known but also beginning to map the fundamental architecture of consciousness itself. This work offers the possibility that beyond pharmacology and beyond pathology lie a deeper truth: the brain is capable of healing itself, if given the chance to see itself anew.

Why It Feels the Way It Feels: Consciousness, Emotion, and the Self

The combined findings suggest that DMT does not simply disrupt consciousness but temporarily reorganizes it. When the brain’s narrative machinery is deactivated and internal control structures loosen, previously inaccessible cognitive and emotional states may become available. This neural reconfiguration helps explain why the DMT experience is often described as ego-dissolving, emotionally intense, or spiritually meaningful [4-6]. Connectivity changes documented by Soares et al. reinforce this idea. DMT appears to enhance the function of networks involved in empathy, self-other processing, and emotional integration [5]. The feeling of unity, interpersonal connection, or profound catharsis reported by many participants likely emerges from this rebalancing of emotional circuitry.

This growing body of evidence supports the idea that psychedelics such as DMT are not chaotic disruptors but potential facilitators of reorganization. The brain appears to enter a temporary state of heightened malleability, with more freedom to explore, reinterpret, and reconfigure its own internal models. This may account for their emerging therapeutic potential in treating conditions characterized by cognitive rigidity or emotional suppression, such as depression, PTSD, and anxiety [3,7]. The notion of DMT as an endogenous modulator of consciousness remains speculative but not without basis. The enzyme INMT, required for DMT synthesis, has been identified in brain tissue and in the pineal gland, raising the possibility of internal DMT production in humans [8]. While its precise function remains unclear, some have proposed roles in dreaming, birth, or near-death states, periods commonly associated with nonlinear time, symbolic imagery, and self-boundary dissolution.

Rethinking the architecture of awareness

The neurophysiological patterns observed during DMT-induced states support a reframing of consciousness as a decentralized and dynamic process, highly sensitive to biochemical modulation. Rather than relying exclusively on top-down control from higher-order cortical networks, the brain under the influence of DMT enters a reorganized configuration in which sensory, emotional, and mnemonic signals take precedence. Functional imaging studies suggest that this reconfiguration is not chaotic but marked by a measurable increase in signal diversity, global connectivity, and reduced functional segregation between brain networks [7,8]. These findings imply that the brain does not simply become disordered during the DMT experience. Instead, it undergoes a temporary shift to a more flexible and integrative state that reveals otherwise latent aspects of cognition and consciousness.

One of the most robust findings from DMT neuroimaging studies is the marked disintegration of the default mode network. The DMN is traditionally involved in autobiographical memory, internal narration, and the maintenance of a coherent sense of self. Its deactivation or functional collapse under DMT appears to correlate strongly with subjective reports of ego dissolution. In such states, individuals often describe a breakdown in self-other boundaries, a loss of narrative identity, or a profound sense of unity with their surroundings. These experiences coincide with increased functional connectivity between typically segregated networks, suggesting a form of global brain integration [6,8]. This altered mode of consciousness reflects a redistribution of cognitive authority from hierarchical cortical systems to more distributed, bottom-up processes.

The REBUS model, which stands for Relaxed Beliefs Under Psychedelics, provides a compelling framework for interpreting these shifts. According to this model, psychedelics reduce the precision-weighting of high-level priors, allowing previously filtered or suppressed bottom-up information to enter conscious awareness [7]. In neurobiological terms, this means that the brain becomes less constrained by habitual predictions and more receptive to raw sensory and emotional input. Under typical waking conditions, these high-level predictions stabilize perception and enforce cognitive coherence. Under psychedelics such as DMT, however, they are loosened, enabling the brain to reprocess unresolved material and explore alternative configurations of thought and emotion.

Importantly, this state of increased entropy is not synonymous with neural noise. Rather, it represents a reorganization of brain dynamics that is structured but unfamiliar. The subjective intensity of the DMT experience, which is marked by vivid imagery, symbolic content, and emotional catharsis, corresponds with specific alterations in network behavior. Subcortical regions such as the thalamus, hippocampus, and amygdala become more dominant in shaping conscious experience [6,8]. These regions are heavily involved in emotional regulation, memory encoding, and sensory integration, and their increased influence during the DMT state may explain the heightened affective and autobiographical quality of the experience. The collapse of DMN activity also creates space for enhanced communication between the visual cortex and limbic structures. This may underlie the complex and immersive visuals commonly reported by DMT users, which often include fractal geometries, archetypal figures, or otherworldly landscapes. These images are not arbitrary. They often carry strong emotional or symbolic significance and may reflect the emergence of subconscious material through newly accessible pathways. Increased connectivity between the amygdala and orbitofrontal cortex, for example, has been observed in both DMT and psilocybin studies, supporting the idea that these compounds enhance affective salience and emotional awareness [5].

This reorganization of network connectivity challenges conventional assumptions about the self. Under ordinary circumstances, the self appears as a stable, enduring construct and a unified agent navigating a coherent external world. Under DMT, this coherence disintegrates. The sense of self becomes fluid, distributed, and context-sensitive. Neurobiologically, this is mirrored by the deactivation of self-referential hubs and the rise of more associative and sensory-dominant circuits. What emerges is a model of consciousness as a dynamic system shaped by the interplay of neuroanatomy, receptor-level signaling, and environmental context.

Crucially, this reorganized state appears to have therapeutic potential. When guided appropriately, the loosening of cognitive constraints may allow for the reprocessing of traumatic memories, rigid thought patterns, or deeply ingrained emotional defenses. This idea is supported by clinical research showing sustained improvements in depression, anxiety, and post-traumatic stress symptoms following psychedelic-assisted therapy [2,5,7]. DMT, in particular, may offer a rapid and intense means of initiating such reprocessing, given its profound effects on both cortical and subcortical systems.

The state of consciousness induced by DMT is temporary, but its effects can be long-lasting. The participants frequently describe these experiences as some of the most meaningful of their lives, often citing newfound clarity, emotional release, or existential insight. This suggests that the changes in brain dynamics under DMT are not merely transient disruptions but may act as catalysts for longer-term psychological integration. The return to baseline brain function after the experience does not erase the narrative or emotional content that emerged. Instead, the restructured experience can be reintegrated into an individual’s broader life story, potentially fostering healing and growth. From a system neuroscience perspective, DMT demonstrates that the brain is not a rigid hierarchy of modules but a flexible, adaptive network capable of profound reorganization under the right conditions. The self, as it is typically experienced, is a construct that depends on the continuous interaction of predictive coding, sensory input, memory, and emotion. When this construct is loosened, as it is under DMT, a new configuration emerges, one that may feel chaotic but is in fact rich with interconnection and potential meaning. In this light, DMT is not just a hallucinogen but a tool for exploring the elasticity of the mind. It allows researchers and participants alike to probe the outer limits of awareness, to observe the brain in a state of radical plasticity, and to question the very assumptions that underlie consciousness itself.

Ultimately, the DMT experience reveals the self as a process rather than an entity, a dynamic unfolding shaped by the coordination of multiple brain systems. It forces a reconsideration of what it means to be conscious, to perceive, and to exist. And in doing so, it opens the door to new models of mental health, personal growth, and human potential, models that embrace not just the stability of the mind but its capacity to change, to reorganize, and to heal.

## Conclusions

DMT offers not just a window into altered states of consciousness. It presents a direct challenge to the structural foundations of the mind. Through the rapid disintegration of the default mode network and the emergence of globally integrated brain activity, DMT reveals a truth that conventional neuroscience often skirts around: the self is not fixed. It is not rooted in any one location. It is a dynamic process, shaped by memory, emotion, identity, and perception, and it can be dismantled and reassembled within seconds.

Ultimately, DMT reveals something profound. Consciousness is not confined. It is responsive. It is structured but never static. It is capable of reorganizing itself in ways that stretch the boundaries of perception and selfhood. The psychedelic experience is not an escape. It is an encounter, and in that encounter lies the possibility of not just understanding the mind but transforming it.
